# A Web Platform for Standardized Data Acquisition, Processing, and Export in the Child Psychopathology Clinical Routine (MedicalBIT): Design and Implementation Study

**DOI:** 10.2196/36757

**Published:** 2022-07-11

**Authors:** Paola Colombo, Silvia Busti Ceccarelli, Stefano Pacchiarini, Stefano Cribellati, Massimo Molteni

**Affiliations:** 1 Child Psychopathology Unit Istituto di Ricovero e Cura a Carattere Scientifico Eugenio Medea Bosisio Parini Italy; 2 SEGE Srl Milan Italy

**Keywords:** digital health, big data, developmental psychopathology, neurodevelopmental disorders, digital data, digital innovation, mental health, screening tool, children, psychopathology, web platform, digital intervention, clinical outcome

## Abstract

**Background:**

The rapid extent of digital innovation for the collection of data has transformed the way in which health professionals collect, share, and analyze health information for better clinical decision-making and health care. In the last decade, there has been an increased interest in telemedicine by mental health agencies; the gap between the need for care and both diagnosis and treatment is wide, and digital technology could play an important role in filling this gap. However, there are limited data on the effectiveness of the clinical process and cost-effectiveness of most telemedicine applications.

**Objective:**

This study examined the implementation of the first Italian online, web-based, comprehensive screening tool and described the screening and diagnostic process through the interactive web platform in a child psychopathology clinic. This is a feasibility study that aims to present the design and implementation of the best practices to improve patient experiences and clinical outcomes. Moreover, the paper evaluates the platform with qualitative and quantitative measures.

**Methods:**

We planned, designed, and implemented a web-based system to collect, store, and manage clinical data. The platform was developed by a multidisciplinary team composed of researchers, clinicians, and informatics professionals through different steps. First, we defined the clinical information to be collected. A number of measures were chosen, tapping several clinical risk areas such as neurodevelopmental disorders and emotional and behavioral problems. The web application architecture and process were then designed. The three phases of process design are described in detail: design of the input interface, processing design, and design of the output interface. Finally, the system has been implemented and evaluated. Based on indicators recommended by the National Quality Forum and the Italian National Guidelines, we evaluated the quality of the system and used quantitative measures that were replicable and comparable over time.

**Results:**

We present the implemented architecture and features of Medea Information and Clinical Assessment On-Line (MedicalBIT), and we provide performance measures for the data collected between October 2018 and June 2021. The measured concepts pertain to four domains: access to care, financial impact/cost, experience, and effectiveness.

**Conclusions:**

In this study, we present the successful implementation of an innovative digital tool. The findings of this study show that the implemented web-based platform appears to be an efficient, cost-effective, and feasible way to improve digital care in the field of child psychiatry.

## Introduction

Digital data collection is an emerging trend in various fields, including the medical and psychological ones. Digital innovation is changing the way health information is collected, shared, and analyzed for better clinical decision-making and health care. Digital innovation is also rapidly expanding in the medical field, significantly improving the quality of health care, reducing health care costs, and enhancing research processes [1]. The rapid evolution of technology has recently promoted web platforms for big data collection in the health care field, and related publications are exponentially increasing [2].

“Big data” refers to a voluminous collection of information taking place quickly without affecting quality, and there are several well-known definitions describing this [3-5]. Pastorino and colleagues [2] stress the importance of defining data as both smart and big because big data presents a substantial potential when it is meaningful. This implies using data for improving health conditions by searching for increasingly clearer and more accurate links between causes, diseases, therapies, and outcomes. In the Study on Big Data in Public Health, Telemedicine, and Healthcare [6], the European Commission identified four macroareas in health care for big data use: (1) early signs for detection, diagnosis, and intervention; (2) identification of risk factors for diseases to improve prevention; (3) enhancing pharmacovigilance and patient safety by communication of real-time information; and (4) improvement in outcome prediction.

All this is possible since a large amount of data is an invaluable resource for epidemiological studies, analyses of general population needs, treatment evaluation, and experimental designs on the target population. If research develops in this direction, it will enable precision medicine that will contribute to optimization of resources: the right care for the right patient at the right time. Therefore, smart use of big data can provide a possible answer to the need for care: socioeconomic and clinical sustainability. In line with this, health technology assessments aim to inform on safe, effective, patient-centered policy-making as well as determine the greatest value for it.

Against this background, telehealth [7] should be seen not only as a complement or alternative to traditional clinical practice but also as an ideal channel to collect and analyze big data. Besides rapid technological progress, COVID-19 spurred an exponential growth in telehealth, and there will be no return to the prepandemic situation according to the American Telemedicine Association [8]. There are several fields of telehealth, just like there are medical specialties. For example, investments in information technology are promoting the development of telepsychiatry. According to Allen [9] and Torous and colleagues [10] one of the most important contributions of artificial intelligence for psychiatry concerns apps, as shown by their innovative project Learn, Assess, Manage, and Prevent (MindLAMP). MindLAMP is a digital platform used both for clinic and research purposes: it receives patient input and aggregates data, it guides reflection, and it helps orient toward treatments. Through the app, patients can input data in an active way (survey, symptom registration, etc) or in a passive way (information collected in the background even if the user is not using the app), and they can receive some advice and mindfulness resources [11]. It is evident that, in the last decade, there has been an increased interest in telemedicine by mental health agencies; the gap between the need for care and possible answers both for diagnosis and treatment is wide, and digital technology could play a fundamental role [12]. In the child and adolescence mental health services, the parents and caregivers of young patients must be involved in the clinical diagnostic process, both as powerful sources of information to be used by clinicians and to obtain a clear understanding about their child’s difficulties. This engagement should be conducted in a comfortable environment for the participants.

Telemedicine seems to be efficient both for the assessment and treatment. For example, among services for the diagnosis of autism spectrum disorder (ASD), the use of telemedicine has shown some promising results in terms of observation [13-15]. As for remote treatment, several recent studies on telemedicine and developmental disorders such as autistic spectrum disorder, specific learning disorder, specific language impairment, dyspraxia, and acquired brain injuries show encouraging results [16-20].

A review of the literature by the National Quality Forum—a no-profit organization for health care development—found a positive effect of telehealth on the quality of processes, outcomes, and costs. It also identified the medical areas where telehealth spread more extensively, with a correspondingly greater increase in scientific publications, namely, dermatology, mental health, rehabilitation, medical management, and chronic diseases. However, despite this topic being current and debated, telemedicine is relatively recent, and there is a lack of studies assessing its quality [21].

In our study, we examined the implementation of the first Italian online, web-based, comprehensive screening tool: Medea Information and Clinical Assessment On-Line (MedicalBIT) [22]. We carried out a feasibility study aimed to present the design and the implementation of the best practices to improve users’ experiences and discuss the evaluation of the platform with qualitative and quantitative measures. Additionally, we intend to describe a web-based screening and diagnostic process in a child psychopathology clinic.

## Methods

### Overview

This section describes the key elements of the platform implementation.

We planned, designed, and implemented a web-based system to collect, store, and manage clinical data through different steps. The whole eHealth system is composed of users (patients and clinicians), a service provider (the Association La Nostra Famiglia-IRCCS Eugenio Medea, a no-profit organization providing care and rehabilitation to children with disabilities), and a service developer (SE-GE Consulting Company).

The main purpose of the MedicalBIT platform is to support diagnostic flow by systematic data collection.

Patients can answer questionnaires comfortably from home through a user-friendly and easily accessible interface, saving time traveling to and staying in a clinic. Clinicians have access to a real-time description of individual patients’ symptoms and a graphical output of possible alerts through clinically based algorithms. Clinicians then assign patients to different diagnostic paths for principal neurodevelopmental disorders (Attention-Deficit/Hyperactivity Disorder, Autism Spectrum Disorder, Specific Learning Disorders, Language Disorders) and behavioral and emotional disorders based on this output. Taking into account the international gold standard, a clinical assessment is performed for each path. The final diagnostic output may differ from the initial path assignment based on the assessment results.

Finally, the data collected can be easily exported in Excel (Microsoft Corporation) format for research analyses: researchers can use collected data for further analysis to develop predictive models.

**Figure 1:**
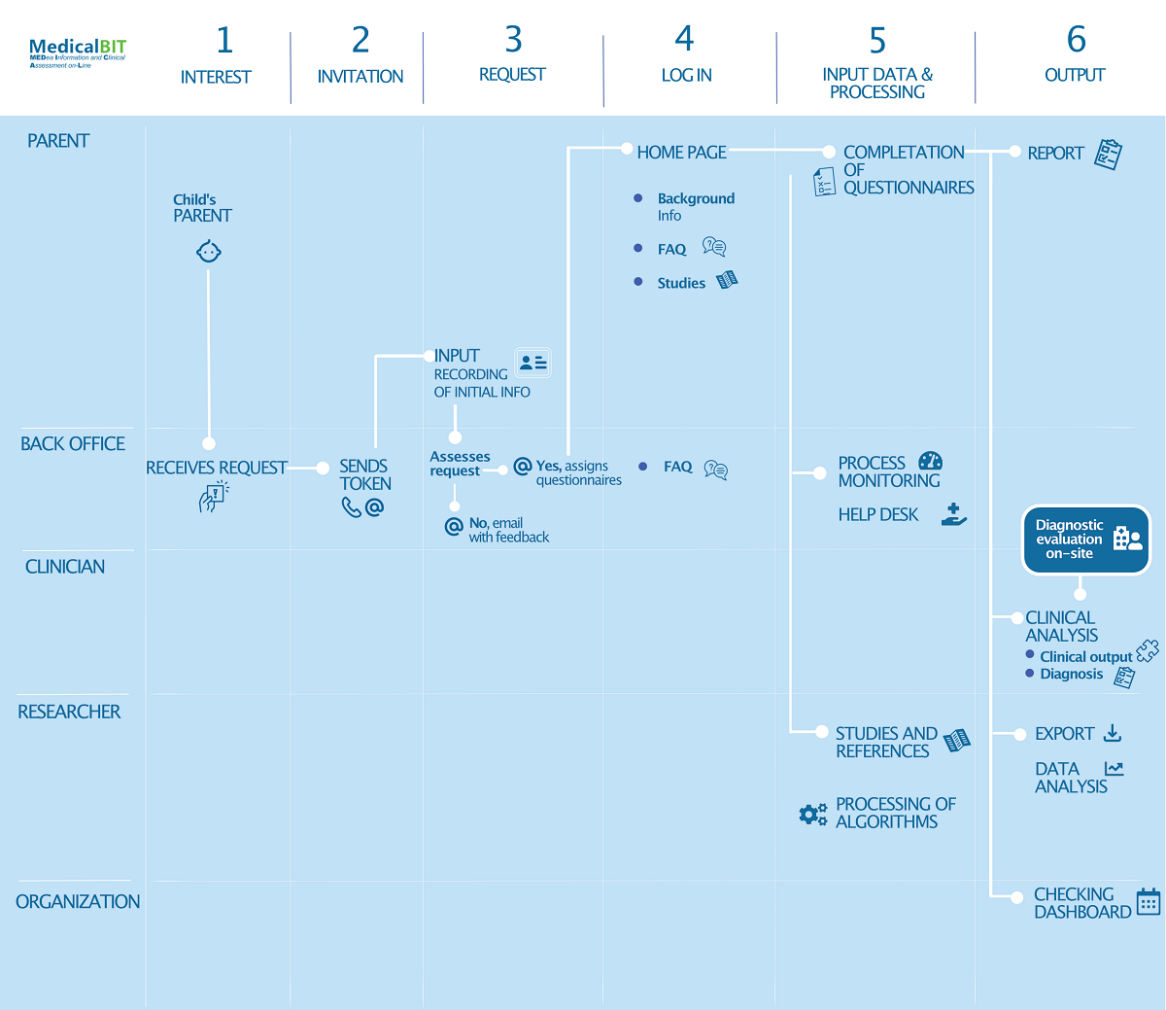
The diagnostic flow on Medea Information and Clinical Assessment On-Line (MedicalBIT).

**Figure 2:**
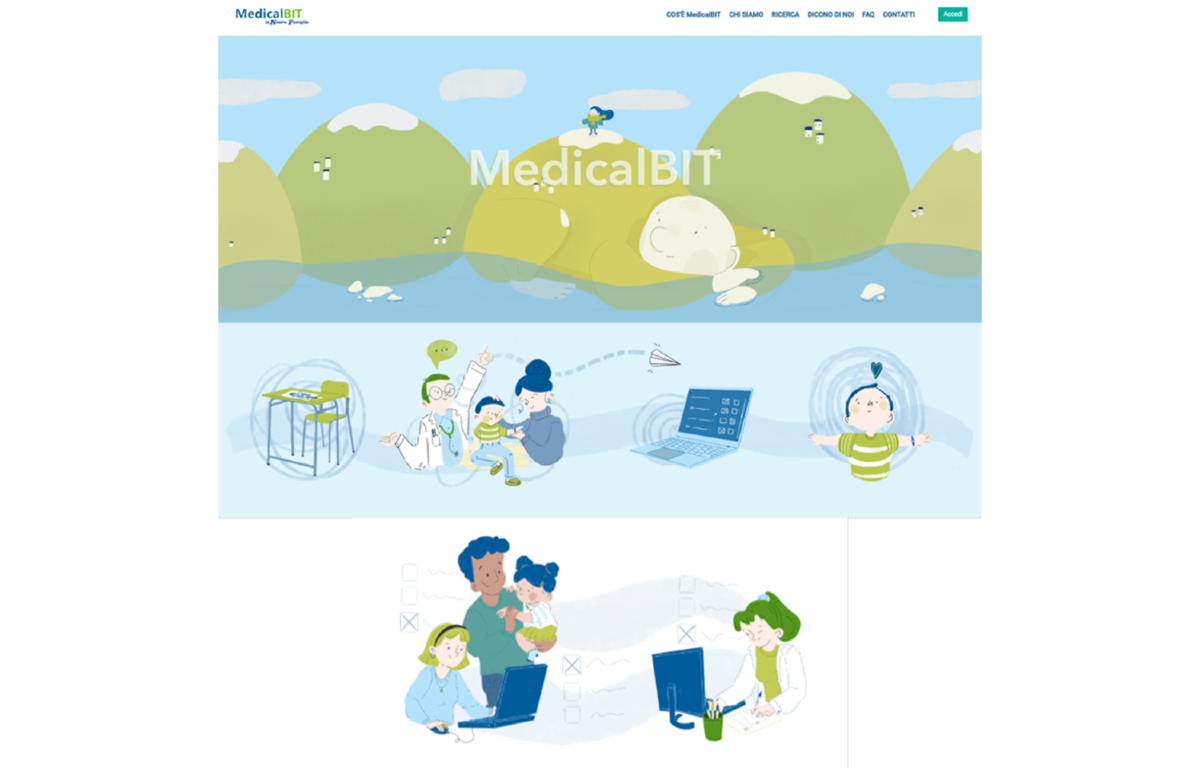
The main menu and some pictures of the Medea Information and Clinical Assessment On-Line (MedicalBIT) home page.

The platform was developed by a multidisciplinary team composed of researchers, clinicians, and informatics professionals. Figure 1 provides an overview of the workflow, and Figure 2 displays some picture frames of the web platform home page. A description of each step is reported below:

1. Defining clinical information to be collected
2. Web application architecture and process design
3. System implementation and evaluation

### Defining Clinical Information to Be Collected

A number of measures were chosen by tapping several clinical risk areas, such as neurodevelopmental disorders and emotional and behavioral problems. Measures were selected for their feasibility in routine clinical practice (ie, brevity, free availability, validation in children and young people, and translation) and psychometric performance (ie, validity, reliability, and sensitivity to change).

The following questionnaires were selected:

- Risk factors: A questionnaire to explore biological and environmental risk factors such as family composition; presence/absence of psychiatric diseases in parents or close relatives; prenatal, perinatal, and postnatal factors; and developmental milestones.
- ASD: The Modified Checklist for Autism in Toddlers [23] and the Autism Spectrum Quotient: Children’s Version [24] were used for the detection of ASD. The first is one of the most widely used ASD toddler screening instruments; it is easily accessible and low-cost. The second is a brief, parent-reported, 50-item questionnaire to quantify autistic traits in children aged 4-11 years.
- Emotional and behavioral problems: The Strengths and Difficulties Questionnaire [25] is a brief instrument widely used to assess main areas of developmental psychopathology and personal strengths. It consists of 25 items and is available in three forms depending on responders: parents, teachers, and adolescents (self-report).
- Other neurodevelopmental disorders: Ad hoc screening tools for specific language impairment and specific learning disorder were implemented by our institute’s research group working on specific learning, language, and communication disorders.

Overall, these tools cover ages ranging from 18 months to 15 years.

After this initial screening step, other standardized assessment tools can be administered too, such as the Child Behavior Checklist (CBCL) [26,27] and the Development and Well-Being Assessment (DAWBA) [28]. Through an interoperability system, links and credentials can be sent to caregivers to complete questionnaires (CBCL thorough ASEBA-Web) and the interview (DAWBA thorough Dawba.net), and results can then be imported by uploading appropriately encrypted files.

### Web Application Architecture and Process Design

**Figure 3:**
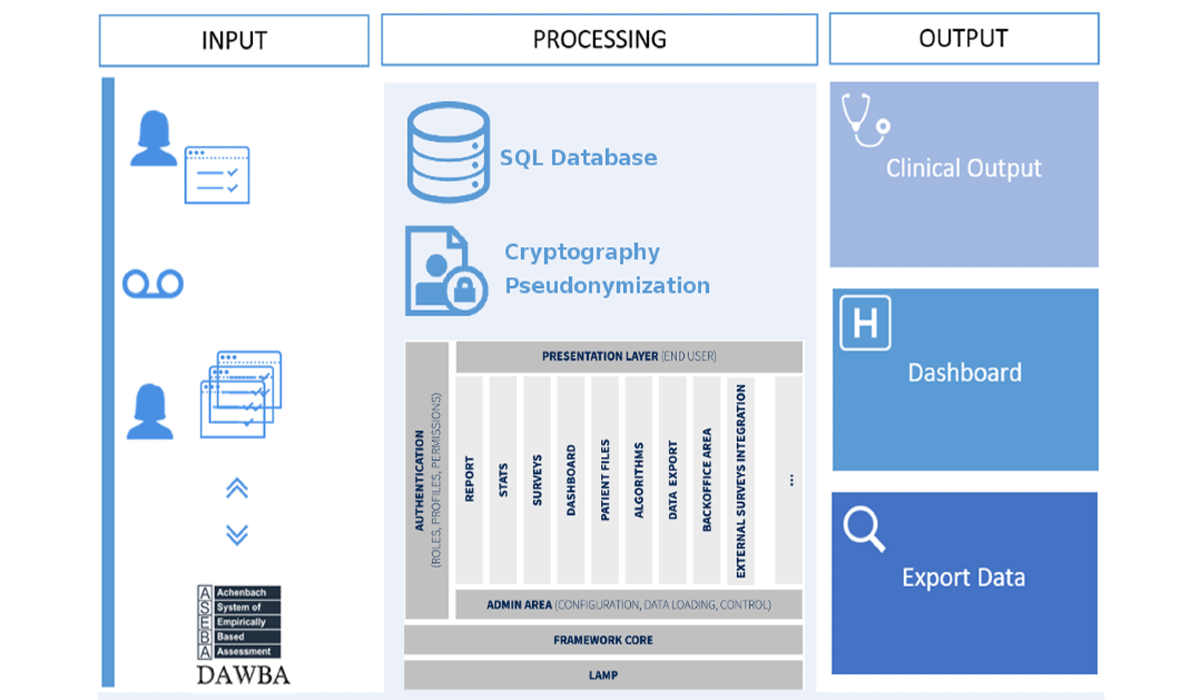
The developed web app architecture design.

Figure 3 shows a high-level diagram of the platform development process. The three phases of process design are described in detail below:

- Design of the input interface
- Processing design
- Design of the output interface

### Design of the Input Interface

An easy-to-use interface was developed to support the interaction between front-end users and the web-based application; the web interface is simple to learn and easy to use, and it displays information in a consistent and progressive manner and maximizes functionalities. To be displayed properly on most mobile devices, the platform implements a responsive web design.

The system also relies on the active engagement of caregivers who send their data to the informatics system using a PC and mobile device. Patients scheduled for an appointment at the Child Psychopathology Unit (IRCCS Medea) get an email with a token for the first web app. They are then guided through a series of steps: completion of a brief form with primary clinical information, verifying of this information, and filling out of the registration form. They then receive a set of questionnaires according to their age and main characteristics, along with the link to the MedicalBIT assessment for completion.

Users are not asked to complete all forms in one session, they can resume from where they last left. After completing all the selected questionnaires, patients are scheduled for a doctor’s visit.

To support the user’s interaction with the platform, a user-friendly back-end interface was implemented. Through a control panel, staff can handle main functions such as administration rights, patients’ lists, and support for data entry.

### Processing Design: Technical Implementation of Data Storage and Automated Scoring Algorithm

At the end of each survey, the system immediately processes the caregiver’s answers using artificial intelligence algorithms. Starting from scores provided for every possible answer and relying on appropriate threshold values, alerts, and key performance indicators are generated, which are useful to health professionals not only to monitor single patients but also for statistics and research purposes.

The whole process is General Data Protection Regulation compliant. The web portal is protected with a digital certificate issued by a certification authority, implements HTTPS (the secure browsing protocol for the World Wide Web), and is hosted on a virtual server in the GARR cloud environment. GARR is the Italian ultrabroadband network for education and research.

The infrastructure provides high reliability, service continuity, and data protection. Backup takes place daily, and we rely on disaster recovery as a service in an alternative environment to ensure availability in case of downtime. We used open source solutions for easier integration with company applications and to avoid vendor lock-in. Our platform is compliant with Italian guidelines [29]: “To ensure an effective support to all company processes, it is necessary to guarantee data univocity and integrity, real-time updating, historicization and audit trail, ergonomics, standardization, integration, stability, availability, security, privacy, innovation, evolvability.”

All data are unique; nothing is duplicated. Information can be accessed only by authorized users and is protected from unauthorized changes; furthermore, confidential data is safeguarded.

There are two different access levels:

- Front-end user
  Caregivers filling out the forms can only view and download the information concerning their child. They have no access to scores generated by the automatic scoring algorithms. Multiple patients can be associated to each caregiver (eg, in the case of siblings).
- Back-end users
  Health care professionals viewing the answers and any alerts enter the diagnoses at the end of the diagnostic process.
  Back-office users with full operation rights
  The system administrator has access to additional features such as dashboard and data export (see next paragraph).

The integrity of data and documents is guaranteed by the use of appropriate logs of activity and changes. These logs are also useful from a legal point of view. All information relating to a particular patient can also be deleted by users with administrator rights. Documents saved on the server are protected by encryption; to comply with the privacy legislation, the information stored in the database is pseudo-anonymized.

### Design of the Output Interface

**Figure 4:**
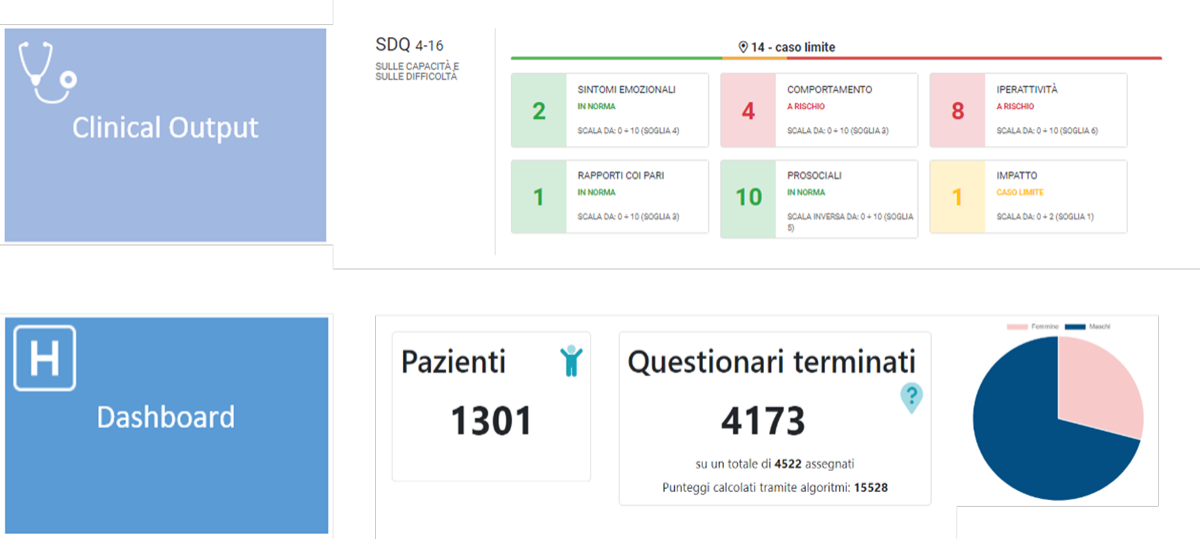
Different output levels.

A real-time graphing, panel, and table interface is available only to back-end users and contains all processed data in real time. This data is also displayed in interactive dashboards available at different access levels. Two different types of reporting are created to display the collected data (Figure 4):

1. Clinical output: an individual-level output, reporting patient outcome scores
2. Dashboard: group-level data, reporting summarized data in a dashboard

All data stored in the database system can be exported in Excel format for further processing.

### System Implementation and Evaluation

This digital system has been on since October 2018 and has been used by 1301 patients (front-end users) and back-end users, including 17 clinicians, 2 back-office operators, and administration operators.

Back-end users are employed by the provider service: back-office operators are office secretaries and administration operators are psychologists and researchers who designed the service in collaboration with the data scientist and consulting company SEGE srl.

Based on indicators recommended by the National Quality Forum [21] and the Italian National Guidelines [29], we evaluated the quality of the system and used quantitative measures that were replicable and comparable over time. As shown in Textbox 1, the selected measures pertain to four areas: access to care, financial impact/cost, experience, and effectiveness.

**Telehealth measurement framework domains and subdomains.**

**Access to care**

- Access for parent/caregiver
- Access to information

**Effectiveness**

- System effectiveness
- Operational effectiveness
- Technical effectiveness

**Costs**

- The financial impact to family/caregiver
- The financial impact to care team

**Experience**

- Patient, family, or caregiver experience

## Results

**Figure 5:**
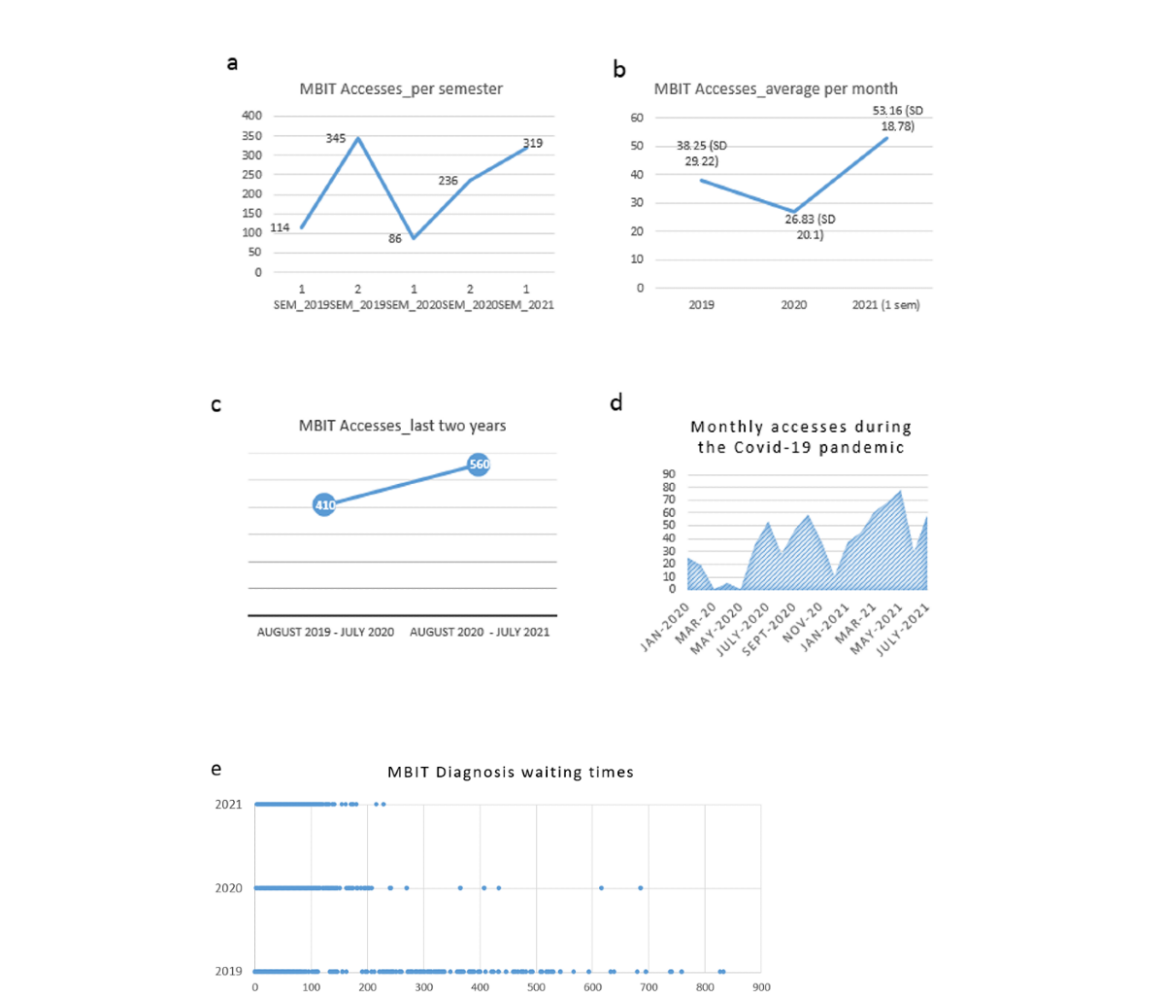
(a) Number of accesses of new patients for each semester in the last 3 years: multiple accesses by 1 patient are counted once. (b) Average and SD per month of accesses by new patients in the last 3 years. (c) Dynamic dimension value = (August 2020 to July 2021) / (August 2019 to July 2020) = 560 / 410 = 1.36. This enabled us to compare differences in accesses in the last 2 years. (d) The graph shows the trend in Medea Information and Clinical Assessment On-Line (MedicalBIT) during the different phases of the pandemic. (e) Data show that the average time between the completion of questionnaires and receiving a diagnosis has clearly decreased over the 3-year period: 2019 (mean 150, SD 179.6), 2020 (mean 82, SD 77.3), and 2021 (mean 65, SD 34.9).

According to the Italian National Guidelines [29] and the National Quality Forum [21], we provide performance measures available for data collected between October 2018 and June 2021 (Table 1 and Figure 5). This process is a work in progress, and in the future, we aim to capture more indicators about different domains and subdomains.

**Table 1:** Platform performance measures.

Domains and subdomains			Qualitative measures		Quantitative measures
**Access to care**					
	Access for parent/caregiver Access to information	The web system is responsive to different devices Family’s patient can access the system using dedicated access points in the institute Clear instructions in the home page Responsive technical assistance		N/A^a^	
**Effectiveness**					
	System effectiveness	N/A		Continuity: from October 2018 to June 2021 Dimension: 1100 new patients accessed the telehealth system and were subsequently sorted to different diagnostic paths. For details about the accesses trend over time, see indicators in Figure 5. Each patient completed on average 4 questionnaires, for a total of 177 items. Speed: (1) Only a few days elapsed between the initial request from family and the completion of screening questionnaires on the platform. (2) The average length between screening questionnaire completion and completion of the diagnostic process was 106 days, with a decrease over the 3-year period from 2019 to 2021 (see Figure 5e)	
	Operational effectiveness	The system is perfectly integrated within the traditional diagnostic path. Customized in-person visits are based on web questionnaire output.		N/A	
	Technical effectiveness	Output dashboard for clinicians and database with collected data for researchers are automatically generated. The system is integrated with other screening and diagnostic tools implemented on different platforms: the Development and Well-Being Assessment [28] and the Child Behavior Checklist [26,27].		N/A	
**Costs**					
	Financial impact for family/caregiver Financial impact for care team	Decrease in the length/frequency of stay/visit to hospital Clinician can integrate traditional work setting with smart working		N/A	
**Experience**					
	Patient, family, or caregiver experience	N/A		Caregivers dropout: (1) 5.3% of registered users do not fill in any questionnaire. (2) 99.6% of caregivers that begin a questionnaire, complete it	

^a^N/A: not applicable.

## Discussion

### Principal Results

In this study, we present the design and implementation of the MedicalBIT web platform, which allows patients to complete questionnaires comfortably from home through a user-friendly and easily accessible interface.

This work is based on the European Commission’s recommendations in the Study on Big Data in Public Health, Telemedicine, and Healthcare [6] that identified four major areas in health care where big data may be used: (1) early signs for detection, diagnosis, and intervention; (2) identification of risk factors for disease to improve prevention care; (3) enhancing pharmacovigilance and patient safety by communication of real-time information; and (4) improvement in outcomes prediction.

The MedicalBIT platform, as described in this paper, allows for the timely collection of clinical data to support the diagnostic process and subsequent steps (1 and 3), and it enables the development of predictive models to improve preventive care and outcome prediction (2 and 4). A core component of the proposed model is caregivers’ perspective of their child’s mental health, laying the foundation for evidence-based practice and person-centered care. The MedicalBIT web platform was introduced into the clinical workflow, making patients’ and caregivers’ care experiences more comfortable. As mentioned, health information technology can save time and reduce costs, thereby improving the health care experience for both patients and clinicians. For this purpose, we also collected customer satisfaction feedback.

Furthermore, performance measures enable us to describe the platform with an objective measurement that allows for comparisons both within the same platform over time and between different web platforms used for similar purposes. According to reports so far, available quantitative data shows general upward trends for platform use from 2018 to 2021 despite the apparent drop during the first months of the COVID-19 pandemic, which required a complete reorganization of care provision.

This trend is in line with research confirming the value of telepsychiatry [30,31] not only during the pandemic period but also before and, most importantly, after the emergency. As described in the Introduction, use of telemedicine in the psychiatry and developmental psychiatry fields can offer several benefits such as improved efficiency and effectiveness of mental health services: it can reach more people with fewer resources [32].

We hope that these changes will lead to filling the care gap in the field of child psychiatry, which has been appropriately defined as “one of the most difficult and crucial challenges of the next decade” [12].

Our project fits into this still uncertain and experimental scenario, with its pros and cons. Telemedicine in the child psychiatry clinical routine can help both patients and health care providers save time and provide them easy access to the care system. Furthermore, because of its fast and advantageous features, it facilitates the hospital and clinic workflow, as processing and automatic output of clinical data enables the collection of large amounts of data for research purposes. Nevertheless, there are some limitations to consider such as the impact of physical distance on human relationships and ethical and coroner issues [29].

Further studies are clearly necessary to establish evidence-based telepsychiatry-specific standards of care, following guidelines proposed by the American Telemedicine Association [33] and the American Academy of Child and Adolescent Psychiatry [34].

### Limitations and Further Development

Based on the current limitations, we are working on a customer satisfaction questionnaire for both families and clinicians to collect feedback to make users feel more engaged in this process and help us as back-office operators to improve services. Moreover, we intend to improve our platform performance measurement tools to obtain more quantitative indicators about the four domains presented in Table 1.

According to Waller and Stotler [35], one of the next steps should be the implementation of a measurement and methodology evaluation of the impact of telehealth on clinical outcomes (in our case, on the diagnostic outcome as an indicator of clinical effectiveness). Our intent is to also implement a platform-based service that could follow and provide assistance to patients after diagnosis (information, psychoeducational materials, rehabilitation tools).

### Conclusions

This study describes the successful implementation of an innovative digital tool. According to our results, the web-based platform appears to be a feasible, efficient, and cost-effective method for enhancing digital care in the field of child psychiatry. It also contributes to the collection of big and smart data, in line with European guidelines. This work shows how large data sets may enhance the accuracy and timing of diagnosis, and contribute to more effective interventions based on predictive models.

## Abbreviations

ASD: autism spectrum disorder
CBCL: Child Behavior Checklist
DAWBA: Development and Well-Being Assessment
MedicalBIT: Medea Information and Clinical Assessment On-Line
MindLAMP: Learn, Assess, Manage, and Prevent
